# Characterization of Rutting Damage by the Change of Air-Void Characteristics in the Asphalt Mixture Based on Two-Dimensional Image Analysis

**DOI:** 10.3390/ma15207190

**Published:** 2022-10-15

**Authors:** Kang Zhao, Hailu Yang, Wentao Wang, Linbing Wang

**Affiliations:** 1 National Centre for Materials Service Safety, University of Science and Technology Beijing, Beijing 100083, China; 2 Sensing and Perception Lab, School of Environmental, Civil, Agricultural and Mechanical Engineering, University of Georgia, Athens, GA 30602, USA

**Keywords:** air-void characteristics, fractal dimension, crack initiation and propagation, rutting damage

## Abstract

In the process of the rutting test, the air-void characteristics in asphalt mixture specimens are a dynamic change process. It is of great significance to systematically study the correlation between the change of air-void characteristics and the depth of the rutting slab and establish a relationship with damage. In this paper, the air-void information of rutting specimen sections with different loading cycles (500, 1000, 1500, 2000, 2500, and 3000 times) is obtained by two-dimensional image technology. The dynamic change process of the micro characteristics of internal air voids of two graded asphalt mixtures (AC-13 and AC-16) under cyclic wheel load is analyzed, and it is used as an index to characterize the microstructure damage of the asphalt mixture. The results show that the variation of air-void distribution, air-void shape characteristics, and air-void fractal dimension with the loading process can well characterize the permanent deformation law of the rutting slab. The fractal dimension of the air void increases with the increase in load. It is a dynamic process in which the air-void content changes with crack initiation and propagation. After rutting deformation, the total air-void area and average air-void size of the sample increase, and the total air-void number decreases. Because microcracks are formed in the specimen after rutting damage, the aspect ratio of the air void increases, and the roundness value decreases.

## 1. Introduction

An asphalt mixture is a composite material composed of aggregates, air voids, mastic, and other multiphase media, which is generally considered a continuous medium. However, the anisotropy of the microstructure leads to complex mechanical states and complex interactions between the components of the asphalt mixture. Among these, the air-void characteristics significantly impact the mechanical properties of the asphalt mixture. We found that two specimens with the same gradation and same air-void content may have different air-void numbers and average air-void size distributions. Specimens with the same air-void content may be composed of air voids of different sizes according to a certain combination. Therefore, two specimens with the same air-void content may also have different failures under the same load conditions. Specimens with larger air voids are more prone to relatively early failure compared with specimens with smaller air-void sizes. This is because once the specimen is subjected to load conditions, the possibility of inducing larger strains increases. In turn, it may lead to internal structural instability, and the existing air voids may continue to expand in the specimen and connect (or merge with other air voids), resulting in serious damage [1]. The increase in air void will reduce the resistance of the asphalt mixture to pavement damage. This is because the air void cannot transfer the load, and the material becomes weaker due to the decrease in the effective area (The concentrated force of each area is higher). The air-void content of the asphalt mixture shall be within a reasonable range to avoid adverse damage such as rutting [2]. Therefore, the proper compaction degree is significant for the performance of asphalt materials. The compaction index is widely used in engineering practice to ensure good service performance of asphalt mixture [3,4].

In recent years, with the progress of technology and the improvement of equipment available, the digital imaging technology of asphalt concrete is an effective tool for evaluating the internal structure. Researchers have been able to use digital image technology to monitor the internal failure process of engineering materials and connect this failure with the measured strain. Internal failure in materials can be represented in many forms, such as specific voids [5], crack surfaces [6,7], and spacing between cracks [8,9]. Air voids play an important role in characterizing the performance of asphalt mixture, and their distribution is very important for determining the overall mechanical response of asphalt mixture [10,11,12]. Under load, existing air voids may merge, resulting in microcracks at the interface between aggregate and mastic. Microcracks continue to expand and grow under deformation, forming macro cracks and increasing air voids [13]. Xu G et al. [14] extracted the internal void structure characteristics of asphalt mixture through an X-ray computed tomography (CT) test and three-dimensional (3D) image reconstruction technology. The change of air-void distribution before and after the freeze–thaw test is analyzed to evaluate the structural evolution of materials under freeze–thaw cycles. Yang B et al. [15] studied the correlation between the performance of porous asphalt mixture and the three pore characteristic parameters obtained by the CT. It was found that the micro pore characteristic parameters had little effect on high temperature, humidity sensitivity, and cooling performance but had a strong correlation with spalling resistance, permeability, connectivity, noise reduction, and other properties. Zhang Z et al. [16] used the connective void content of three types of asphalt mixtures was employed to characterize the damage to the corresponding asphalt mixture sample under freeze–thaw cyclic loading. The variation of connective void content revealed the nonlinear characteristics of asphalt mixture damage accumulation. Kassem Emad et al. [17] used several mechanical tests (the overlay tester, Hamburg Wheel-Tracking Test (HWTT), and a repeated tensile test) to characterize the influence of air-void distributions on mechanical properties and response of asphalt mixtures. The results show that air voids play an important role in influencing the performance of asphalt mixtures. Xu H et al. [18] used a set of image analysis programs to extract the internal structural characteristics of asphalt mixture during freeze–thaw cycles. The evolution of the internal void structure of the asphalt mixture during the freeze–thaw cycle was evaluated. Hassan et al. [19] analyzed the air void and crack characteristics caused by stress and strain by using a two-dimensional (2D) image analysis method and took them as the damage index to characterize the microstructure damage of the asphalt mixture. Therefore, it is very meaningful to analyze the damage behavior of asphalt mixture by describing the change characteristics of air voids in the deformation process of samples. However, previous researchers used image analysis to characterize the void of asphalt mixture, focusing on the change value before and after the test and less on the development of the whole process of void during the test. At the same time, the change of air-void content is more used as an evaluation indicator, and other intuitive and effective indicators are lacking. Few studies have used the rutting test to characterize the change in the internal voids of materials.

The purpose of this paper is to quantify the change of air-void characteristics as a damage characterization method by analyzing the section image information of rutting specimens during the test. This method can not only be used to quantify the changes of air-void characteristics in the process of damage development but also to determine the damage concentration area and use the determined air-void parameters to describe the severity of the damage. Although most of the current research involves 3D analysis, the internal structure of materials is obtained through various advanced scanning techniques. It is also important to understand the basic 2D measurement in damage analysis because it can provide a reference for specific problems. Using the same concept, further analysis of damage can be extended to complex problems in 3D or four-dimensional (4D) analysis [20]. At present, most of the research focuses on the change of meso-void characteristics of the whole specimen before and after wheel loading. Few studies have explained the air-void distribution of the whole wheel load area section in the whole process of loading and linked the air-void distribution with the change of air-void fractal dimension and permanent deformation of the asphalt mixture. This study is a comprehensive study on the variation law of internal air-void characteristics of two kinds of asphalt mixtures (AC-13 and AC-16) with the loading cycles of the rutting test. It is the basic work to explain the evolution of internal defects of mixtures under wheel load and evaluate the high-temperature performance of asphalt mixtures from a meso perspective.

## 2. Materials and Methods

### 2.1. Materials

In this study, the dense gradation, namely AC-13 and AC-16, with the nominal maximum aggregate size of 13 and 16 mm, was selected. Qinhuangdao 70# asphalt was selected as an asphalt binder, and the main physical properties are given in Table 1. The coarse aggregate is limestone, the fine aggregate is machine-made sand (0–5 mm), and the filler is limestone powder. Asphalt mixture samples are prepared under the optimum asphalt binder content obtained by the Marshall method [21]. The aggregate gradation, optimum asphalt binder content, and air-void content of the two asphalt mixtures are shown in Table 2. The air-void content of all types of samples is controlled within ±0.5% of the range shown in Table 2.

### 2.2. Specimen Fabrication and Rutting Test

**Figure 1:**
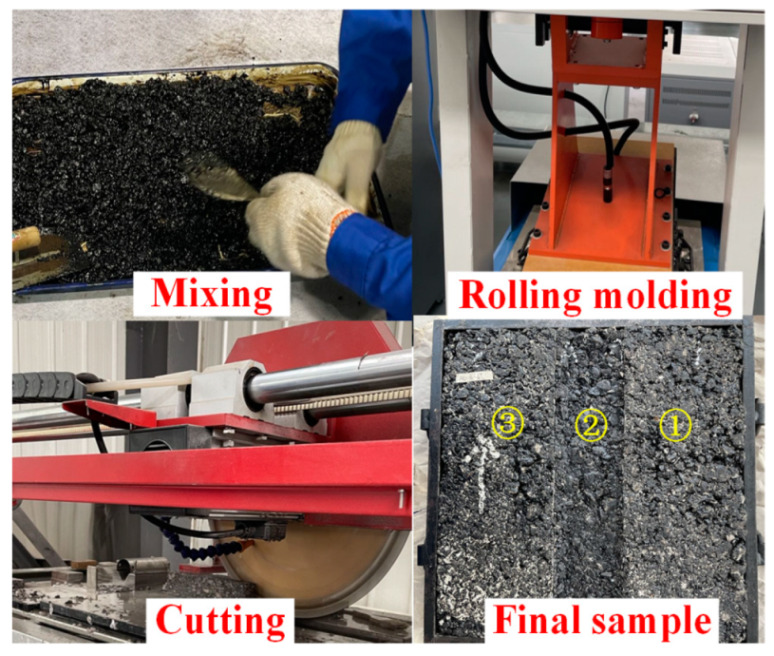
Specimen preparation and cutting.

According to the Chinese code [22], the mixing temperature of asphalt mixture containing petroleum asphalt is 163 °C. The compaction temperature of the rutting specimen is 100 °C during the forming process. Therefore, the rolling equipment and test mold shall be preheated to 100 °C in advance. The roller load is set as 9 kN (line load 300 N/m). The manufacture of a rutting specimen consists of two steps: (1) rolling the rutting specimen for two cycles with a roller (moving back and forth on the sample is defined as one cycle) and (2) rolling the sample for 12 cycles after rotating it 180°. The size of the final formed rutting specimen is 300 (length) × 300 (width) × 50 mm (height). In order to accurately obtain the change of specimen section after different loading cycles, the plate is cut into three parts by an asphalt mixture cutter along the wheel loading direction before the test, 60 mm in the middle and 120 mm on both sides. Therefore, 3 specimens named 1, 2, and 3 are obtained, as shown in Figure 1.

**Figure 2:**
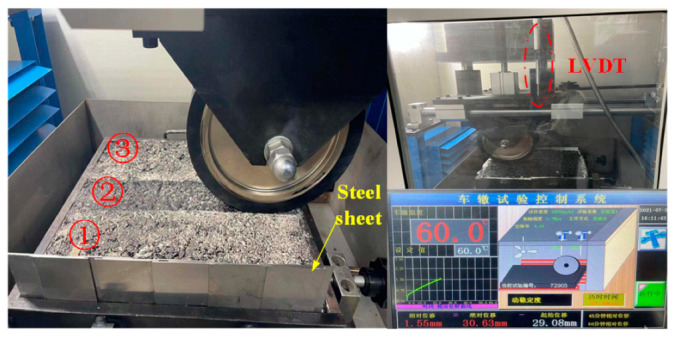
Rutting test.

The rutting test is carried out by using the automatic asphalt mixture rutting instrument to study the rutting development of the asphalt mixture. The device is mainly composed of rubber wheels and a fixed platform. The diameter of the rubber wheel is designed to be 200 mm, and the contact width of the wheel specimen is 50 mm. The stress applied by the rubber wheel is 0.70 ± 0.05 MPa, and it is loaded back and forth on the rutting specimen at the rolling speed of 42 r/min. The experimental temperature is set at 60 °C [22]. Due to the cutting effect, the overall size of the rutting specimen is reduced by 4 mm. In order to keep the boundary conditions unchanged, 4 mm steel sheets are inserted on one side to provide a constant lateral force. The details of the rut test are shown in Figure 2. The cumulative rutting depth changes of the specimens under different loading times (500, 1000, 1500, 2000, 2500, and 3000) are recorded by the linear variable differential transformer (LVDT) in the device. In this study, 3 rut specimens of AC-13 and AC-16 are used, respectively.

### 2.3. Image Acquisition and Processing

**Figure 3:**
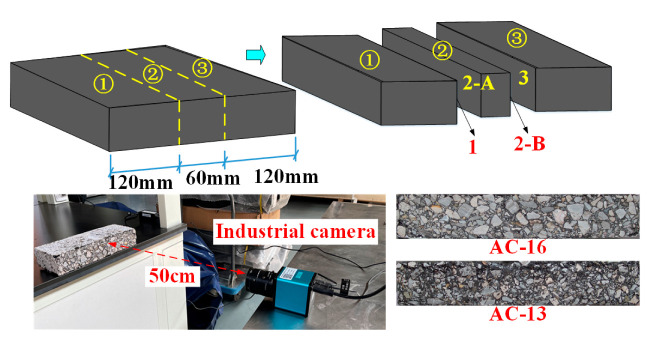
Specimen section image acquisition process.

The industrial camera is used to collect the images of four sections of each specimen after the corresponding loading times (500, 100, 1500, 2000, 2500, and 3000) to study the rutting deformation law. Because of the large difference in each specimen section, we only collected the image information of one of the three samples of each asphalt mixture. When taking images, the camera is placed on the test bench to ensure the levelness of the camera. The camera lens is located at the center of the specimen, 50 cm away from the specimen, as shown in Figure 3.

**Figure 4:**
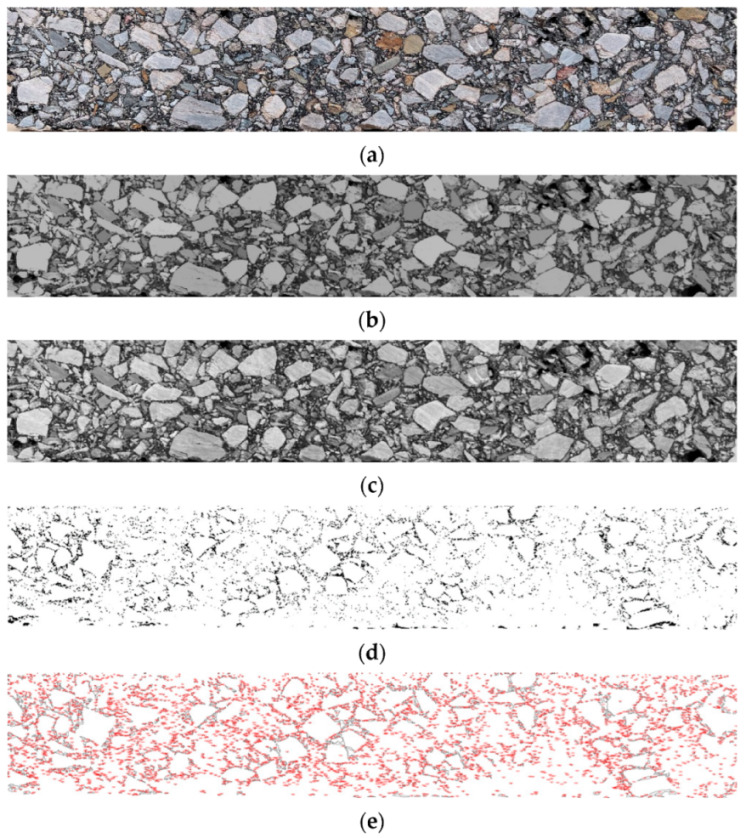
Image processing process: (**a**) original image; (**b**) section image after gray histogram equalization; (**c**) section image after median filtering; (**d**) sectional image after image segmentation; (**e**) air-void feature information.

Digital image processing uses a series of algorithms to process and analyze digital images with computers so that the images can meet the needs of human vision, other equipment, data extraction, etc. [23]. In this section, MATLAB software is used to process images, including image enhancement, image filtering, image segmentation, and feature information acquisition. (1) Image enhancement: image enhancement is mainly processed by histogram equalization, also known as gray-level equalization. The purpose is to convert the input image into an output image with the same number of pixels at each gray level through the operation of each pixel in the image. The cross-section image of the asphalt mixture specimen after gray histogram equalization is shown in Figure 4b. Histogram equalization function in MATLAB is histeq (f, 256). (2) Image filtering: In this study, median filtering method is used to filter the image, which is very effective for filtering pulse interference and image scanning noise. The cross-section of the specimens after median filtering is shown in Figure 4c. The function of the median filtering operation in MATLAB is medfilt2 (f). (3) Image segmentation: This research obtains the best threshold value through the function gray threshold value in MATLAB [24]. According to the function result, when the threshold value is set to 35, a good segmentation effect can be obtained, as shown in Figure 4d. Figure 4e shows the acquisition of air-void feature information on the segmented image using ImageJ software. The whole process is shown in Figure 4. By comparing with the air-void content of the actual mixture, we selected the air void when the intensity of the corresponding pixel is in the range of 0 to 20. The air voids are divided into small air voids (<1 mm^2^), medium air voids (1–5 mm^2^), and large air voids (>5 mm^2^) according to the size.

### 2.4. Air-Void Characteristics

Some parameters, such as air-void content, air-void number, air-void shape, and average air-void size, have been successfully used to characterize the characteristics of air voids. Among them, the number and average size of air voids are very important in characterizing the characteristics of air voids. In the study of microstructure damage, the changes of these parameters (by comparing the parameters during and before deformation) provide information on the damage degree of rutting specimens with different gradations, different depths, and different loading accumulation times.

**Figure 5:**
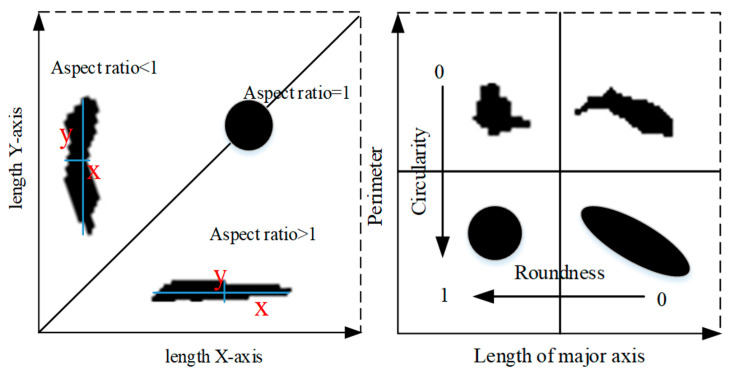
Shape features.

In 2D images, the shape is usually regarded as the area surrounded by a closed contour curve. The most commonly used shape factors in image analysis are roundness, circularity, and aspect ratio. Figure 5 shows air-void shape features defined according to roundness, circularity, and aspect ratio. The shape factor is usually normalized, ranging from 0 to 1. A shape factor equal to 1 usually represents the ideal case or maximum symmetry, such as a circle and sphere [25]. Generally speaking, the crack has a high aspect ratio, and the circularity and roundness values are low (close to 0.0), showing a flat shape. In this study, the section information of the specimen before and after the rutting test is obtained through image analysis. The average air-void size, aspect ratio, circularity, and roundness of the air void in the whole process of the test are calculated by using Equations (1)–(4) to study the change of the air void.

(1) $$\mathrm{Average}\;\mathrm{air}\;\mathrm{voids}\;\mathrm{size}=\frac{\mathrm{Total}\;\mathrm{air}\;\mathrm{void}\;\mathrm{area}}{\mathrm{Number}\;\mathrm{of}\;\mathrm{air}\;\mathrm{void}},$$

(2) $$\mathrm{Aspect}\;\mathrm{ratio}=\frac{\mathrm{Length}\;\mathrm{of}\;\mathrm{major}\;\mathrm{axis}}{\mathrm{Length}\;\mathrm{of}\;\mathrm{minor}\;\mathrm{axis}},$$

(3) $$\mathrm{Circularity}=\frac{4\pi \times \mathrm{Area}}{{\mathrm{Perimeter}}^{2}},$$

(4) $$\mathrm{Roundness}=\frac{4\pi \times \mathrm{Area}}{\pi \times {\mathrm{Major}\;\mathrm{axis}}^{2}},$$

### 2.5. Fractal Dimension Theory

**Figure 6:**
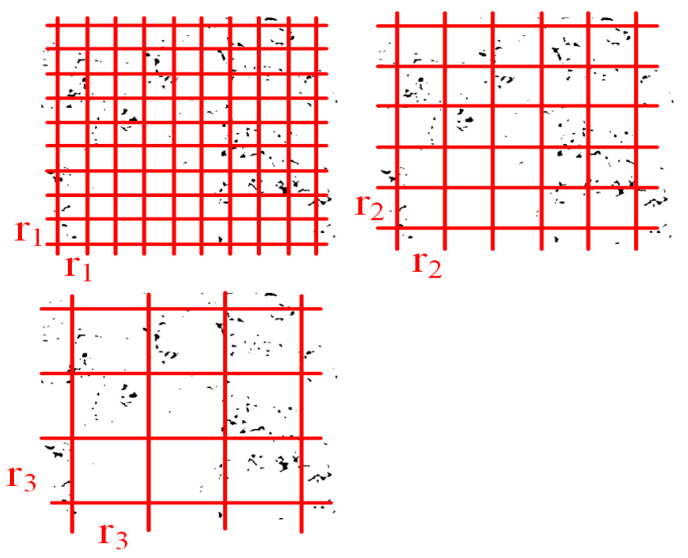
Images of box arrays of different sizes (r).

**Figure 7:**
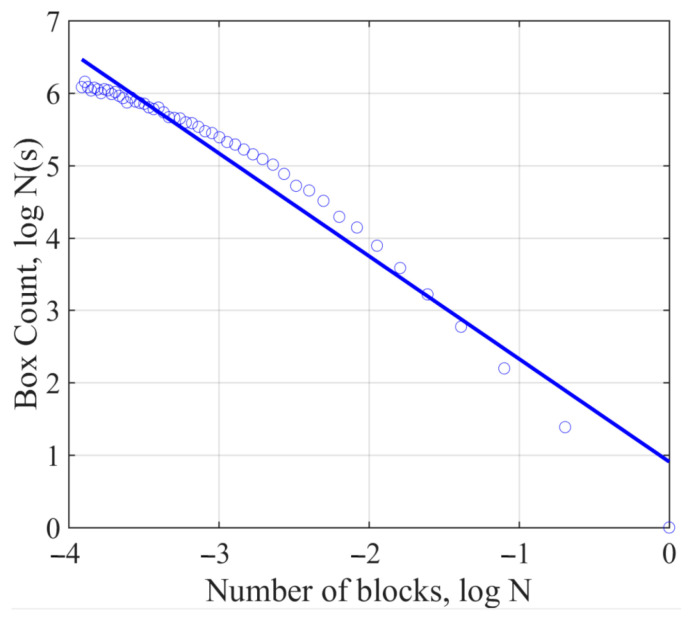
Logarithmic relationship between box count and each box size.

The fractal dimension reflects the validity of space occupied by complex objects. It is a measure of the complex shape and irregularity of objects, including Hausdorff dimension, box-counting method, etc. [26]. Box counting uses a set of square boxes or grids to measure the length or distance between points on the shape boundary. It can be calculated with MATLAB software [27]. Figure 6 shows an image of a box array of different sizes (r) covering the air-void image. In the curves of Log (box number, N) and Log (box size, r), the boxes containing the pixels of the air-void image are counted (N), which is used to obtain the fractal dimension, i.e., the slope of the logarithmic regression line. As shown in Figure 7, it can be seen from the fitting curve of Log N (s)–Log N that the image shows the self-similar characteristics of the fractal body at different scales. This shows that the fractal dimension based on 2D images can well reflect the changes in micro-structure. Therefore, it is feasible to quantitatively characterize the rut section by fractal dimension. We can use Equation (5) to calculate the fractal dimension. Lower fractal dimensions represent more stable air-void structures. If the measured air-void areas are the same, different air-void distributions will produce different fractal dimensions.

(5) $$FD=-\frac{\log(N(r))}{\log(r)},$$

## 3. Results

### 3.1. Correlation between Rutting Performance and Air-Void Characteristics

#### 3.1.1. Rutting Test Results

**Figure 8:**
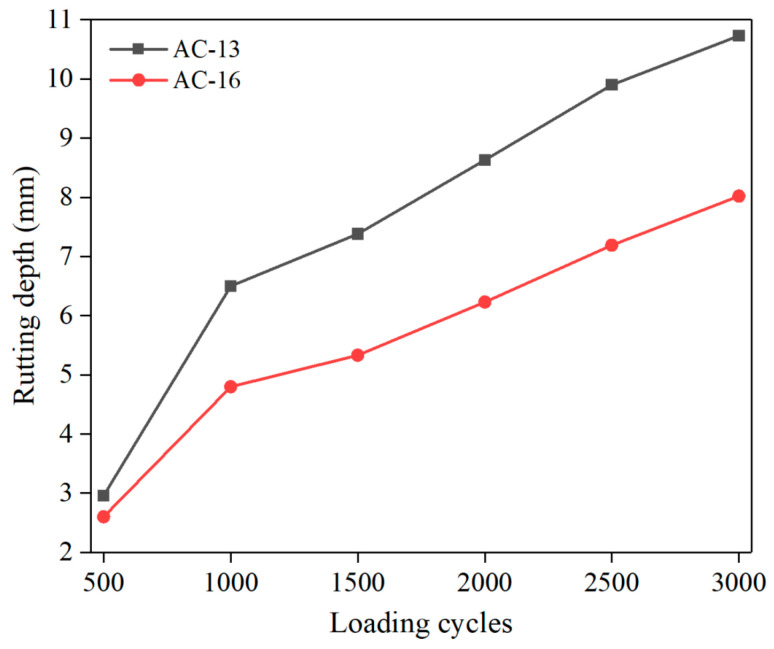
Rutting test results.

Rutting depth is an important index to evaluate the rutting resistance of specimens. The average value of rutting depth measured on different specimens (three samples for each grading) of the same grading is taken as the rutting test results of this study. The rutting test results are shown in Figure 8. As shown in Figure 8, the deformation of AC-16 is less than that of AC-13 during the whole flow deformation. Different asphalt mixture gradation leads to different deformation development of rutting specimens. Later, we try to explain this phenomenon from the damage analysis of the screenshot image of the rutting specimen.

#### 3.1.2. Correlation between Rutting Cumulative Depth and Air-Void Characteristics

**Figure 9:**
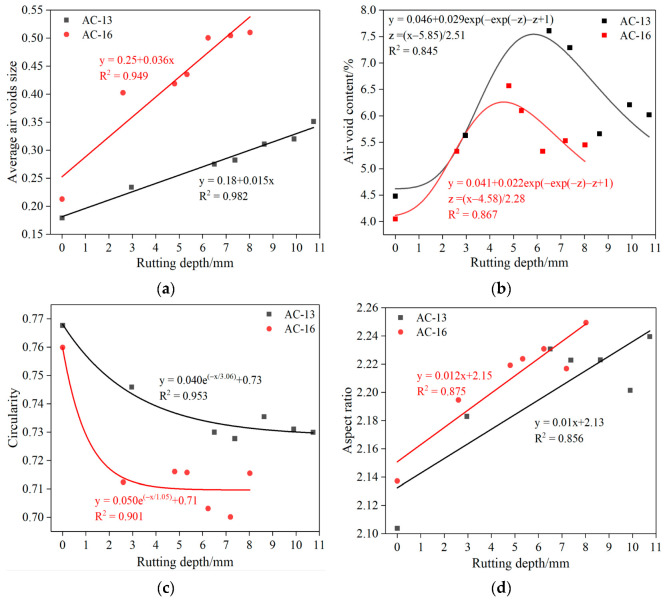
Correlation between rutting depth and air-void distribution: (**a**) average air-void size; (**b**) air-void content; (**c**) circularity; (**d**) aspect ratio.

Previous studies have found that the damage of the asphalt mixture under load may first occur in its internal air voids [28]. Therefore, it is of great significance to deeply understand the permanent deformation of the specimen by studying the variation characteristics of the air voids in the specimen during the rutting test. The correlation between the accumulated rut depth and the air-void characteristics (average air-void size, air-void content, circularity, and aspect ratio) is shown in Figure 9. In order not to increase the influence of other factors, only the air-void characteristics of section 2-A after different loading times are studied here.

The cumulative rut depth is linearly related to the average air-void size and aspect ratio, as shown in Figure 9a,d. With the increase in the average air-void size and aspect ratio, the cumulative rut depth also increases. At the same time, according to Figure 9c, with continuous loading, the air void inside the specimen becomes flatter, and the circularity decreases. At this time, the internal damage of the specimen increases, and it is more prone to damage. It can be seen from Figure 9b that with the increase in cumulative rut depth, the air-void content first increases, then decreases, and finally tends to a stable value. This is mainly because the number of air voids will first increase rapidly under the load. With the increase in load, the air voids in the specimen are continuously compacted and merged into larger air voids, finally forming cracks. Therefore, the air-void content decreases to a certain extent. This is consistent with Ma X et al.’s research on two possible air-void evolution laws in the mixture [29]. This indicates that under load, the change of internal air voids, along with the initiation and propagation of cracks, is a dynamic process, which is of great significance to the study of internal damage of rut specimens.

#### 3.1.3. Correlation between Rutting Cumulative Depth and Air-Void Fractal Dimension

**Figure 10:**
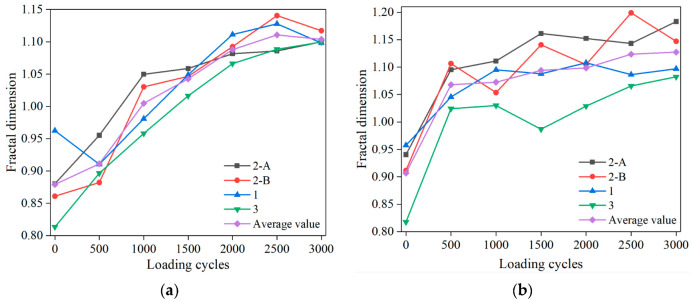
Relationship between fractal dimension and loading cycles: (**a**) AC-13; (**b**) AC-16.

**Figure 11:**
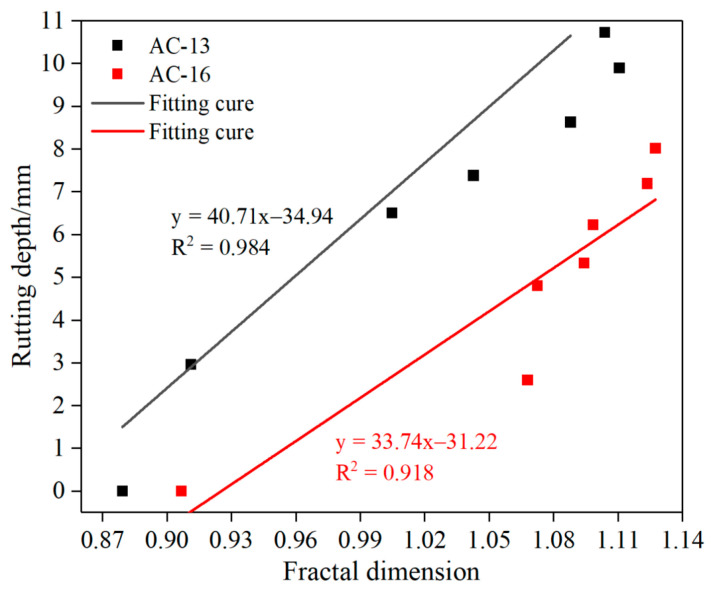
Relationship between fractal dimension and rutting depth.

Based on the fractal theory, the relationship between the fractal dimension of air voids and the rutting depth of rutting specimens is studied. It solves the difficulty of measuring the deformation of each layer in the sample by a conventional test method. The measured fractal dimensions of the AC-13 and AC-16 sections vary with the loading cycles, as shown in Figure 10. For the relationship between fractal dimension and rutting depth, we only analyze the fractal results of section 2-A, and the results are shown in Figure 11. The fractal dimension is related to the complexity and disorder of surface morphology, and more complex image surfaces have higher fractal dimensions [30]. In general, the fractal dimension of air voids increases with the increase in loading times. In other words, with the increase in loading times, the air-void complexity increases. This is consistent with the formation of many irregularly shaped air voids and the increase in roughness in the specimen section during the test.

By studying the correlation between rutting depth and air-void fractal dimension, it is found that the value of the fractal dimension is linearly correlated with rutting depth. Therefore, fractal theory can reliably study the deformation law of rutting samples. With the development of deformation, the fractal dimension of cross-section air voids increases gradually. During the test, air voids of all sizes are compacted under the wheel load and create new air voids, especially in the area below the load. With the development of rutting depth, new air voids appear, and the old air voids fuse and merge into micro cracks, which increases the complexity and dispersion in the section. Obviously, the fractal results of air voids accord with the physical significance of fractal theory.

### 3.2. Air-Void Structure and Rutting Specimen Damage

#### 3.2.1. Change of Air-Void Content

**Figure 12:**
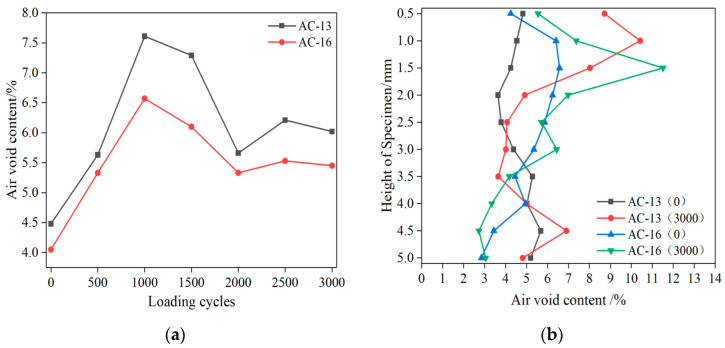
Change of air-void content: (**a**) change with loading cycles; (**b**) along the depth direction.

In order to characterize the internal damage of the material by the change of the air-void content of the specimen. The changes in the air-void content of the 2-A section of the two mixtures with the loading cycles and the height along the specimen before and after loading are studied, respectively, as shown in Figure 12.

The air-void content of the two rutting specimens first increases, then decreases, and finally stabilizes with the increase in loading cycles, as shown in Figure 12a. This is mainly due to the formation of a large number of air voids in the loading area under compression and shear at the early stage of loading. The newly formed air voids are more than the air voids used for fusion and forming microcracks, so the air-void content will have a rising process. With the increase in loading cycles, a part of the internal air void of the rutting specimen is fused and merged to form micro cracks under load, and a part of the air void is compacted, so the internal air-void content will have a decline process. In the last stage, damage occurred inside the specimen. The change process of air voids is similar to the previous two stages, but this process is not as violent as before. Finally, the air voids will gradually tend to a stable value. Due to the lack of laboratory technology to accurately capture the air-void distribution of asphalt mixture, previous research results are based on the air-void content before and after the test [31]. It is of little significance to study the damage development process.

It can be seen from Figure 12b that before and after the test, the area with high air-void content change mainly occurs in the middle and upper part, which indicates that under the action of wheel load, this area is subject to more shear deformation, resulting in more new air voids. For AC-13, the air-void content in the middle area of the section decreases after the test. Because this area is mainly subjected to compressive stress and the air voids are continuously compacted, the air voids are reduced. The increase in bottom air-void content indicates that it is subjected to shear stress. For AC-16, the change of air-void fraction in the middle area is not obvious, and there is a certain decrease in the bottom. This is mainly due to the large particle size of aggregate in AC-16 and the good performance of the skeleton in the middle area, which can withstand large shear stress failure.

#### 3.2.2. Damage Analysis of Rutting Specimen

**Figure 13:**
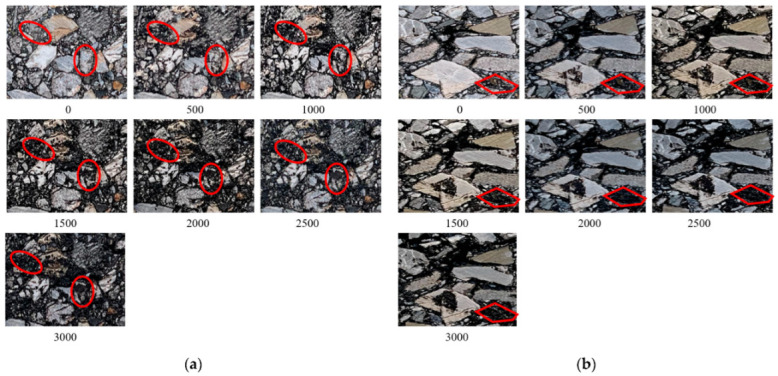
Variation of sectional air void with loading cycles: (**a**) AC-13; (**b**) AC-16.

Previous studies have shown that the load transfer capacity of asphalt mixture mainly depends on the interaction between aggregate and mortar, and macro cracks appear at the interface between aggregate and mortar [32]. Figure 13 shows the change process of air-void shape of the two mixtures under wheel load and also vividly represents the formation process of macro cracks in the mixture. As shown in Figure 13b, under the action of wheel load, the air void develops along the interface between aggregate and asphalt mortar. This process leads to the deformation, expansion, and fusion of the air void and promotes the generation of macro cracks in the mixture. Micro damage also promotes the bond failure or damage of asphalt mortar, further has a more adverse impact on the bonding performance between aggregate and asphalt mortar, and aggravates the degradation of internal structure under a wheel load. In addition, it can be seen from Figure 13a that the load also makes the aggregate with smaller particle size in the mixture crushed, which destroys the internal skeleton structure of the mixture and further accelerates the damage of the asphalt mixture. Comparing the two mixtures, it can be seen that this phenomenon is more obvious for the mixture with a smaller maximum particle size of aggregate. The damage is not only the increase in air void but also accompanied by the crushing of many aggregates and the overflow of asphalt mortar. This is different from Li P et al.’s result that only aggregate movement exists in asphalt mixture under pressure [33], which provides a new idea for the study of mixture damage.

#### 3.2.3. Change of Air-Void Characteristics

**Figure 14:**
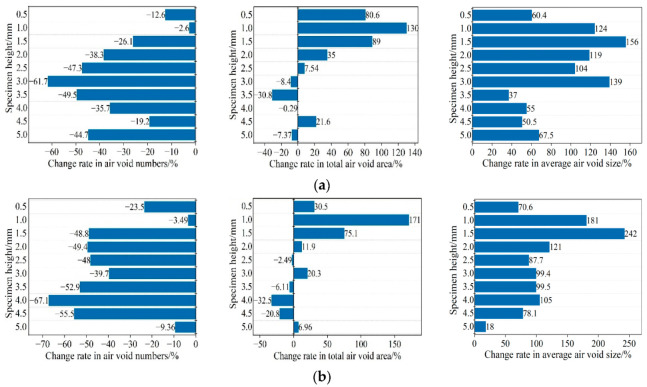
Variation of air-void characteristics in rutting test: (**a**) AC-13; (**b**) AC-16.

Figure 14 shows the changes in total air-void number, total air-void area, and average air-void size of the two graded mixtures before and after the rutting test. These parameters have been successfully applied to characterize the characteristic changes of voids and have been well-established in previous research work [34,35]. In the study of microstructure damage, the changes of these parameters (by comparing the parameters before and after deformation) provide valuable information on the severity of damage at different depths in compacted asphalt mixture specimens. The bar graph compares changes in air-void characteristics by averaging at 5 mm intervals along the height of the specimen. Through observation, several noteworthy results were obtained along the characteristic distribution of the sample height in three main areas, namely, the bottom (0–1.5 cm), the middle (1.5–3.5 cm), and the top (3.5–5 cm).

As shown in Figure 14, the rate of change of total air-void area and average air-void size is higher in the middle and upper region, which is consistent with the slight expansion observed in the middle and upper region of the specimen after the rutting test. Under the action of wheel load, the middle and upper mixture is seriously damaged. The total air voids of the two mixture specimens along the height direction are reduced, indicating the generation of micro cracks and macro cracks in the rutting specimen, which also promotes the expansion in the middle area of the specimen. This is mainly due to two reasons: one is that the internal air void is reduced due to the continuous compaction of the specimen under load; Second, microcracks are formed due to the fusion of small air voids.

**Figure 15:**
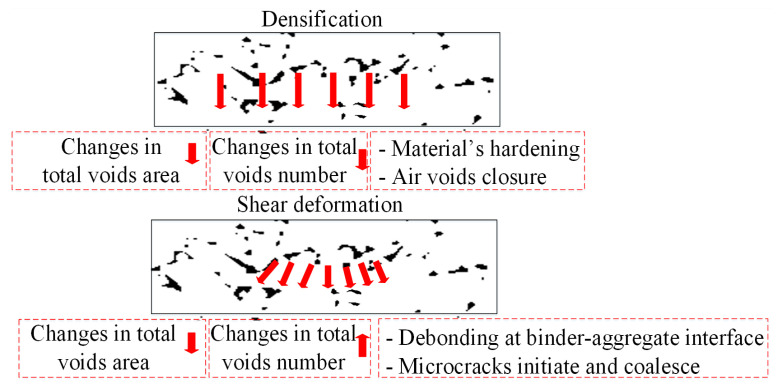
Correlation between damage mechanisms and changes in air-void content properties.

In this study, when describing the different damage phenomena in the specimen due to deformation, the changes in total air-void number and total air-void area are determined as two key parameters. According to the previous study [36], there are two main mechanisms under permanent deformation, namely densification, and shear deformation, as shown in Figure 15. By analyzing the changes in air-void number and air-void area before and after the test, the results of this experiment are mainly related to the damage mechanism related to compression deformation. The relationship between damage mechanisms and air-void properties will be further clarified in subsequent sections, involving changes in air-void geometry.

The change distribution of air-void characteristics along the height of the rutting specimen before and after the test is systematically studied in the previous part. This part will study the change of air void under load from the whole section. Firstly, the variation characteristics of air-void perimeter and area in the mixture under different loading cycles are studied. Secondly, the changes in air voids with different area sizes and their corresponding quantities before and after loading are studied. Finally, the changes in the total area and number of air voids of different types (large, medium, and small) before and after the test are studied.

**Figure 16:**
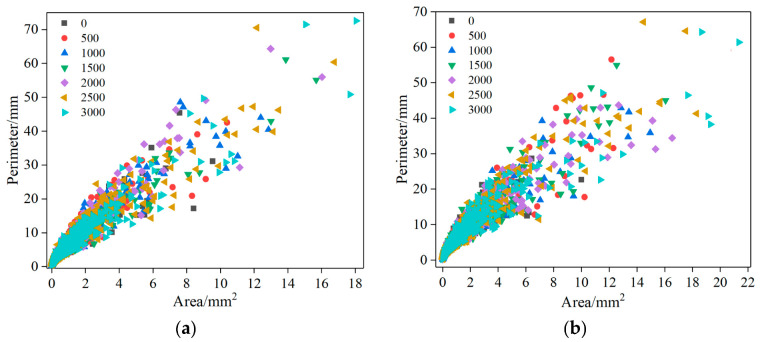
Relationship between air-void area and perimeter: (**a**) AC-13; (**b**) AC-16.

Figure 16 shows the area and perimeter distribution of the air void in the rutting specimen under different loading cycles. When the polygon area is certain, the larger the ratio of major and minor axes, the longer the circumference [37]. It can be seen from Figure 16 that with the increase in loading times, the area and perimeter distribution of the internal air voids of the rutting specimen become wider, and the discreteness also increases. This shows that under the action of load, the air voids develop towards a flatter shape, preparing for further integration and macro cracks. This also confirms the rationality of the previous research on the development law of air voids before and after loading from another perspective.

**Figure 17:**
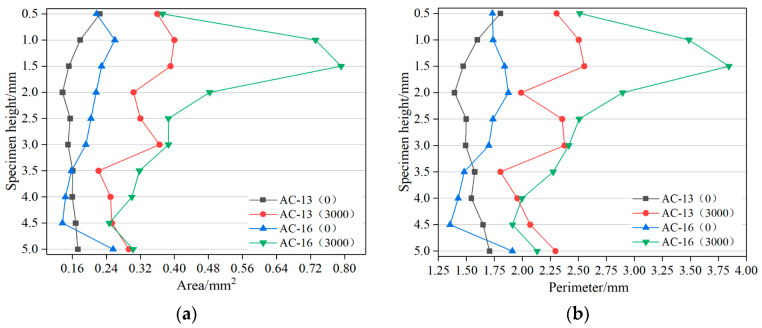
Variation of the average area and perimeter of air voids along specimen height (**a**) Average area; (**b**) Average perimeter.

Figure 17 shows the variation law of the average area and perimeter of air voids along the height of the specimen. It can be seen from Figure 17 that before the load is applied, the average perimeter and area of the two graded mixtures change a little along the height direction, which shows that the specimen is fully compacted during the preparation process. After 3000 cycles of cyclic loading, the average area and perimeter of the specimen increase to a certain extent along the height direction, indicating that the internal air-void size of the specimen increases as a whole. For the two mixtures, the change rate of the middle and upper part of the specimen is large, which is also consistent with the obvious expansion of the middle and upper part observed after the test.

**Figure 18:**
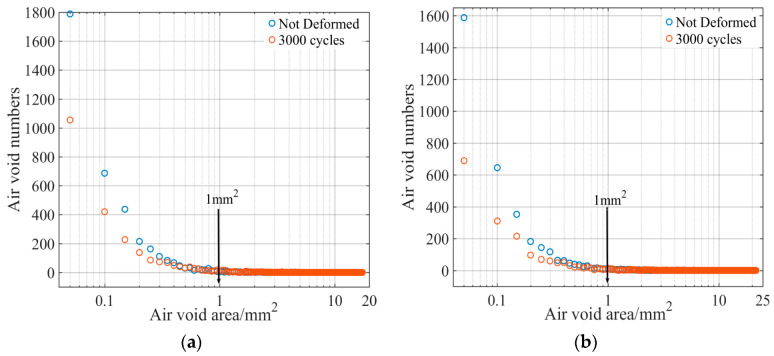
Relationship between air voids and corresponding quantities: (**a**) AC-13; (**b**) AC-16.

**Figure 19:**
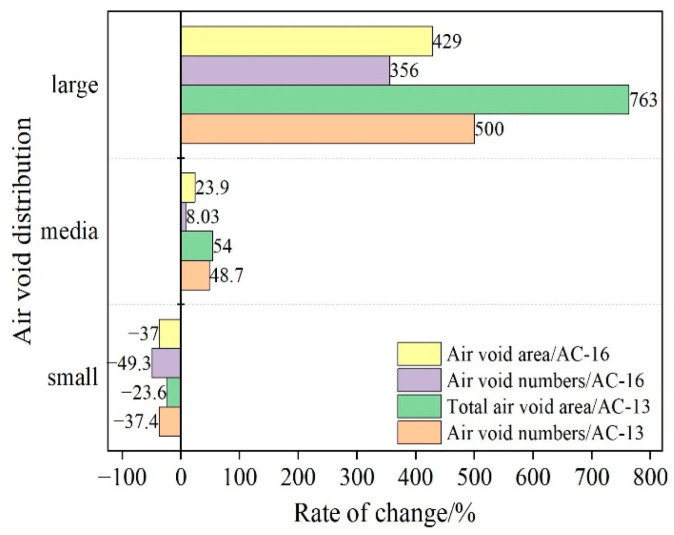
Changes in area and quantity of air voids with different sizes.

In order to further explain the change of air voids before and after the test, we calculated the area and the corresponding number of air voids in the 2-A section of the specimen before and after loading, as shown in Figure 18. Previous field tests have concluded that air voids in the pavement are compressed after permanent deformation [38]. However, it can be seen from Figure 18 that a larger area of air voids appeared in the specimen after loading. The number of air voids with smaller areas (≤1 mm^2^) is reduced greatly, and most of them are compressed. At the same time, we also compared the change rate of the total area and quantity of different types of air voids before and after the test, as shown in Figure 19.

It can be seen from Figure 19 that after 3000 cycles of wheel loading, the total number and area of small air voids in the specimen decrease, while the middle air voids and large air voids increase, and the change rate of the total number and area of large air voids is the largest. This is mainly due to the small number of large air voids in the original specimen. After loading, the number of large air voids increases, making the change rate larger. The above results show that the air voids inside the specimen mainly occur through compaction and fusion of small air voids under load. The fusion between small air voids expands into micro cracks, which in turn induces damage to the asphalt mixture. At the same time, the change rate of the total number and area of the large and medium air voids in AC-13 specimens is higher than that of AC-16, indicating that the damage degree of AC-13 specimens is higher than that of AC-16. This result can be confirmed by rutting results.

#### 3.2.4. Change of Air-Void Shape Characteristics

**Figure 20:**
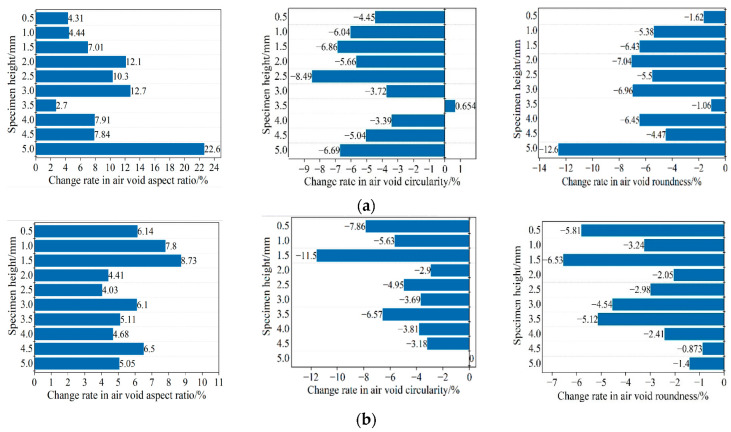
Variation of air-void shape characteristics in rutting test: (**a**) AC-13; (**b**) AC-16.

After the rutting test, not only the total number of air voids, total air-void area, and average air-void size but also the shape characteristics of air voids will change greatly. Figure 20 shows the change rate of void shape characteristics of the specimen before and after the test, i.e., aspect ratio, circularity, and roundness. The bar graph compares the changes in air-void shape characteristics by averaging at 5 mm intervals along with the height of the specimen.

It can be seen from Figure 20 that after the test, the overall roundness of the air voids in the specimen decreased, and the shape of the air voids developed into a flat shape. At the same time, with the increase in air-void size, two adjacent air voids merge to form an air void, and its aspect ratio is higher than that of its single air void. This confirms the previous findings about the change in air-void properties. In general, the method of measuring the shape characteristics of air voids provides a different method to describe the characteristics of air-void formation, growth, connectivity, and expansion. The damage is due to the coalescence of small air voids to form microcracks, which increases the aspect ratio of air voids, thereby reducing the number of air voids and roundness [39]. Based on the observation of a large number of samples, it is determined that the damage controlled by the change of air-void structure occurs in many ways. First of all, these changes may be due to the increase in the existing single air-void size and the propagation of cracks. Secondly, the two separated air voids may merge and expand, forming cracks near the damage. Third, due to deformation, new air voids with crack initiation potential are also formed. This finding is consistent with the two failure types determined by Kim et al. [40]. The damage in the asphalt mixture is the result of microcracks generated in the interfacial transition (ITZ) between asphalt and aggregate and within the binder (cohesive failure).

## 4. Conclusions

This paper aims to quantify the change of air-void characteristics as a method to characterize damage by analyzing the cross-sectional image information of rutting specimens during the test. Firstly, the indoor rutting test was carried out, and the air-void information of the sample interface under different loading times was obtained by digital image technology. Then, the variation law of the internal air-void characteristics of the asphalt mixture with the loading times of the rutting test is systematically studied, and the relationship with the development of rutting is established. Finally, the air-void characteristic parameters are used to quantify the damage in the asphalt mixture and describe the generation and development of damage. According to the research results, the following conclusions can be drawn:

(1) In the process of the rutting test, the change of air-void ratio in the rutting specimen is a dynamic process with the initiation and propagation of the crack. With the increase in cumulative rut depth, the air-void ratio increases first, then decreases to a certain extent, and finally tends to a stable value.
(2) The fractal dimension of air voids increases with the increase in loading times. In other words, with the increase in loading times, the air-void complexity increases. This is consistent with the observation that the section of the specimen expands, and the roughness increases during the test.
(3) In the rutting test, the damage is not only the compaction of voids but also accompanied by the crushing of aggregates and the overflow of asphalt mortar. At the same time, this phenomenon is more obvious for the asphalt mixture with a smaller maximum particle size of aggregate.
(4) The measurement of air-void characteristics and shape characteristics provides a different method to describe the characteristics of air-void formation, growth, connectivity, and expansion of voids in asphalt mixture during a rutting test. After deformation, the total air-void area and average air-void size of the specimen increase, and the total air-void number decreases. Because microcracks are formed in the specimen after rutting damage, the aspect ratio of the air voids increases, and the roundness value decreases.

Therefore, we can use the change of void ratio, void characteristics, and void shape characteristics to quantify the damage type and its evolution process in the rutting formation of the asphalt mixture in future research. Due to the limitations of the technology used in this study, the voids discussed are obtained based on two-dimensional images. It is difficult to distinguish the connection and closure of voids in the three-dimensional case of a mixture, so further research is needed. In addition, in order to determine the main cause of damage to the inner cup of the asphalt mixture, it is necessary to analyze the damage at the contact between aggregate and asphalt and the impact of the contact point between aggregates on the performance.

## Figures and Tables

**Table 1:** The properties of asphalt binders.

Parameter	Qinhuangdao-70#	Requirements	Test Value	Test Method
Penetration (25 °C, 5 s, 100 g) 0.1 mm		60~80	64	T0604
Penetration index (PI)		−1.5~+1.0	−0.32	T0604
Ductility (10 °C)		≥20	42	T0605
Ductility (15 °C)		≥100	>100	
Softening point TR&B/℃		≥46	48.0	T0606
Solubility/%		≥99.5	99.72	T0607
Flash point/°C		≥260	282	T0611
Density (15 °C)		Measured	1.037	T0603
Thin Film Oven Test (TFOT)	Mass loss/%	≤±0.8	−0.177	T0609
	Penetration ratio/%	≥61	65.4	T0604
	Ductility (10 °C)/cm	≥6	9.8	T0605

**Table 2:** Aggregate gradations and mix design results of AC mixtures.

Sieve Size (mm)	Passing Percent (%)	
Gradation	AC-16	AC-13
19	100	-
16	95	100
13.2	85	94.8
9.5	71.2	81.5
4.75	35.4	41.2
2.36	23.9	27.6
1.18	20.4	23.3
0.6	16.2	18.2
0.3	13.0	14.2
0.15	11.1	12.0
0.075	7.8	8.0
Optimum asphalt content (%)	4.6	4.8
Air-void content (%)	4.0	4.4

## Data Availability

Not applicable.
